# Molecular Mechanisms and Regulatory Factors Governing Feed Utilization Efficiency in Laying Hens: Insights for Sustainable Poultry Production and Breeding Optimization

**DOI:** 10.3390/ijms26136389

**Published:** 2025-07-02

**Authors:** Zhouyang Gao, Jiangxia Zheng, Guiyun Xu

**Affiliations:** College of Animal Science and Technology, China Agricultural University, Beijing 100193, China; gaozhouyangcau@163.com

**Keywords:** laying hens, feed utilization efficiency, sustainable agriculture, microbial ecosystems, molecular mechanisms, molecular breeding technologies

## Abstract

Since the early 2000s, the poultry industry in our nation has steadily progressed towards a larger scale and increased intensification. However, the growing demand for animal-based protein, combined with significant increases in feed ingredient costs, presents substantial challenges to the advancement of egg production. The regulation of feed utilization efficiency in laying hens is a complex process, influenced by various factors including the farming environment, feed composition, microbial ecosystems, and hormonal dynamics. The feed conversion rate in laying hens not only serves as a critical indicator of agricultural productivity but also highlights the significant impact of molecular technologies in improving feed efficiency. These technological advancements have enhanced the precision and effectiveness of breeding practices while providing substantial support for optimizing feed management, improving production metrics, and promoting sustainable agricultural practices. This comprehensive synthesis of factors, regulatory pathways, and cutting-edge molecular methodologies establishes a biological framework for future breeding strategies. Notably, this review uniquely emphasizes the pivotal role of modern molecular biology techniques—such as genomic selection, transcriptomic profiling, and gene-editing tools—in decoding feed conversion efficiency (FCE), contributing to broader goals of agricultural sustainability by balancing productivity gains with eco-friendly and cost-effective egg production.

## 1. Introduction

China leads globally in both laying hen and egg production. Currently, the industry aims to extend the production cycle of laying hens to 100 weeks, with the goal of improving the sustainability and efficiency of egg production [1]. However, this extension presents significant challenges. In intensive farming systems, laying hens exhibit high metabolic rates during peak production, but as they age, they frequently develop metabolic disorders. These include the decreased synthesis of yolk precursors in the liver, excessive fat accumulation in hepatic and abdominal regions, the disruption of the body’s redox balance, and the exacerbation of conditions such as fatty liver disease and osteoporosis. As organ functions progressively decline and degenerative diseases emerge, prolonging the production cycle further compromises the performance of aging hens. This manifests in a marked decline in egg production rates, deterioration in egg quality, and reduced overall poultry production efficiency, all of which directly impact the economic viability of poultry farming [2].

Poultry production efficiency is a metric that quantifies the labor input required to produce a unit of poultry products (e.g., eggs or meat) [3]. In comparison to developed nations, China’s poultry production has not yet achieved significant economies of scale. However, as the socio-economic landscape evolves, enhancing poultry production efficiency has become increasingly crucial. A key factor in this efficiency is feed utilization, as feed costs constitute 60–70% of total poultry farming expenses [4]. Consequently, feed utilization efficiency has long been a critical determinant in the economic viability of poultry production. The global increase in prices of feed ingredients such as corn and wheat has further emphasized the necessity of improving feed utilization efficiency and reducing production costs in the livestock industry [5]. As a result, leveraging genetic advancements and modern molecular biology to comprehend and enhance poultry production efficiency remains a primary focus for breeders.

Feed efficiency in laying hens denotes the relationship between feed consumption during growth and egg production quantity [6]. The most common utilized indicators for measuring feed utilization efficiency in production are the feed conversion ratio (FCR) and residual feed intake (RFI). The FCR is defined as the ratio of feed weight consumed to output product weight over a specific measurement period; in laying hens, it is termed the feed-to-egg ratio [7], while in broilers, it is referred to as the feed-to-meat ratio [8]. Studies have reported that the FCR is a quantitative trait controlled by multiple quantitative trait loci, exhibiting moderate heritability, with estimates ranging from 0.12 to 0.49 [9,10]. Variations in heritability estimates across studies may stem from factors such as chicken breed, sex, age, diet, housing conditions, and sample size [11]. Research has demonstrated that selecting for the FCR within breeding populations may lead to increased body size during later growth stages [12,13], thereby increasing the energy required for individual bird maintenance. The costs associated with maintaining body size account for approximately 60% of feed-related expenses. For instance, the FCR shows a strong negative correlation with daily weight gain, indicating that individuals with high feed utilization grow faster [14]. Additionally, as the FCR is a ratio of two indicators, the differing variances of consumption and output result in a non-normal distribution of FCR [15], complicating the acquisition of accurate averages and variance statistics, thereby impeding the selection process in breeding [16]. The concept of RFI was introduced by Mahmud et al. [17], defining it as the difference between the actual feed intake of livestock and the expected intake needed for maintenance and growth demands. Phenotypically, RFI differs from other production traits, with its average value in a population approximating zero [18]. Breeders aim to increase feed utilization efficiency through negative selection based on RFI. Research [16,19] indicates that the heritability estimates of RFI range from 0.20 to 0.45, also classified as a trait with moderate heritability. RFI is often analyzed in correlation with the FCR within the same studies, and biostatistical research has demonstrated a very high genetic correlation between the two traits (0.70–0.94) [6]. Furthermore, studies by De Verdal et al. have shown that selection based on the FCR affects gastrointestinal traits [12], where RFI primarily influences the upper digestive tract, while the FCR mainly affects the lower digestive tract. Related research has found that due to the relatively minor phenotypic correlation of RFI with traits like egg production and weight gain in laying hens, using RFI as an indicator of feed utilization efficiency enables breeders to reduce feed intake significantly without altering overall production performance, thus effectively reducing production costs [20]. In poultry production, the advantage of using RFI as a selection criterion lies in its independence from production traits, demonstrating moderate heritability that can be effectively passed on to offspring [21]. Therefore, compared to the FCR, RFI serves as a more suitable and broadly applicable measure of feed utilization efficiency in livestock.

**Figure 1:**
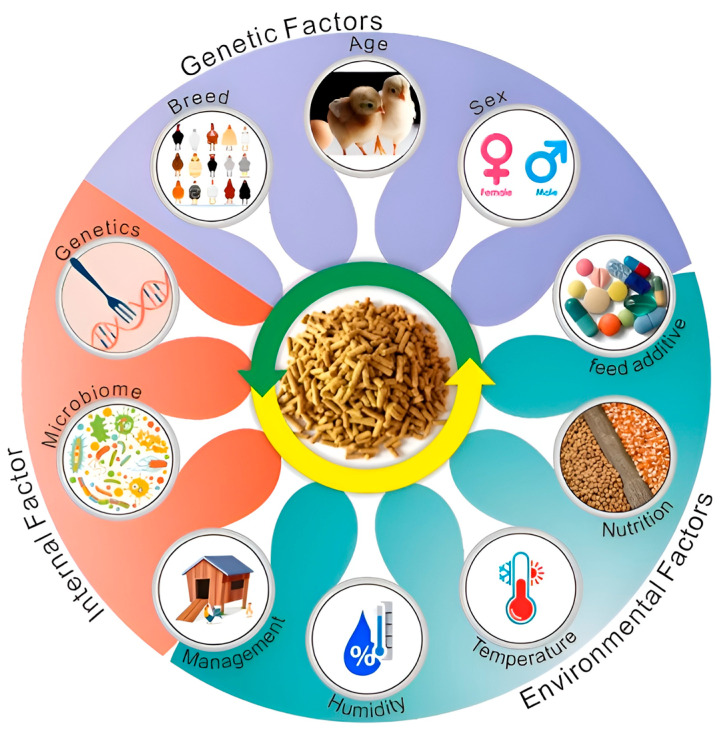
The main influencing factors of feed efficiency in laying hens. Genetic factors: poultry breed, age, and sex. Environmental factors: feed additive level, nutritional level, chicken house temperature, humidity, and management. Internal factors: gut microbiota and genetic regulation.

Currently, feed utilization efficiency is a crucial economic trait for extending the laying period [22], yet research on this trait in laying hens remains relatively limited. This characteristic is influenced by both host genetic factors and the gut microbiota. To achieve the goal of extending the laying period, researchers must investigate the regulatory mechanisms underlying feed utilization efficiency to address the issue of reduced feed utilization in the later stages of egg production. With advancements in modern biological technologies, molecular breeding can significantly impact the feed utilization efficiency of laying hens through various mechanisms, including optimizing gene selection, metabolic regulation, gut microbiota management, nutritional adjustments, and enhancing stress resistance. This approach provides novel strategies for improving the production performance of laying hens and promoting sustainable development. Consequently, this paper systematically examines the influence and regulatory mechanisms of genetic, nutritional, and environmental factors, as well as microbial communities, hormonal regulation, and health status, on feed utilization efficiency in laying hens (Figure 1).

## 2. Factors Affecting Feed Efficiency and Regulatory Mechanisms of Laying Hens

### 2.1. Effect of Feeding Behavior on Feed Efficiency and Regulatory Mechanisms

Feeding is a fundamental process for digestion and absorption in laying hens, serving as the foundation for their growth, development, and production performance. As both FCR and RFI calculations depend on the critical parameter of feed intake, its influence on feed efficiency in laying hens is particularly significant. Feed intake typically refers to the weight of feed consumed by hens over a specified period and is influenced by various factors, including genetics, nutrition, management, and environment [22]. Consequently, feed intake traits are generally not used as direct selection traits for improvement. However, as an essential primary trait, feed intake remains a fundamental indicator for assessing the health status of a population. The feed intake of laying hens closely correlates with their production levels, nutritional health, and feed conversion rates [23]. When feed intake is too low, a greater proportion of nutrients is allocated to maintenance rather than production, resulting in the underutilization of the hen’s production potential and reduced feed conversion efficiency. Conversely, excessive feed intake may not only diminish feed efficiency but also adversely affect lipid metabolism and overall health in hens. Therefore, appropriately reducing feed intake without compromising egg production performance can increase the proportion of feed available for production while improving feed conversion rates.

Current research on feed intake traits in laying hens primarily focuses on elucidating regulatory mechanisms from a biological perspective. The feeding behavior of poultry is regulated by a complex network of neural pathways, influenced by both the central and peripheral nervous systems, encompassing short-term and long-term regulatory mechanisms [24]. The central nervous system plays a crucial role in regulating feed intake; it integrates various food signals and stimulates hens to experience satiety or hunger, thereby controlling the initiation and cessation of feeding [25,26]. The hypothalamus is the core area for regulating feeding in poultry [27], comprising five nuclei: the arcuate nucleus (ARC), paraventricular nucleus (PVN), ventromedial nucleus (VMN), dorsomedial nucleus (DMH), and lateral hypothalamic area (LHA). Through their interactions, these nuclei generate signals related to satiety and hunger, modulating feeding behavior in response to peripheral stimuli.

**Figure 2:**
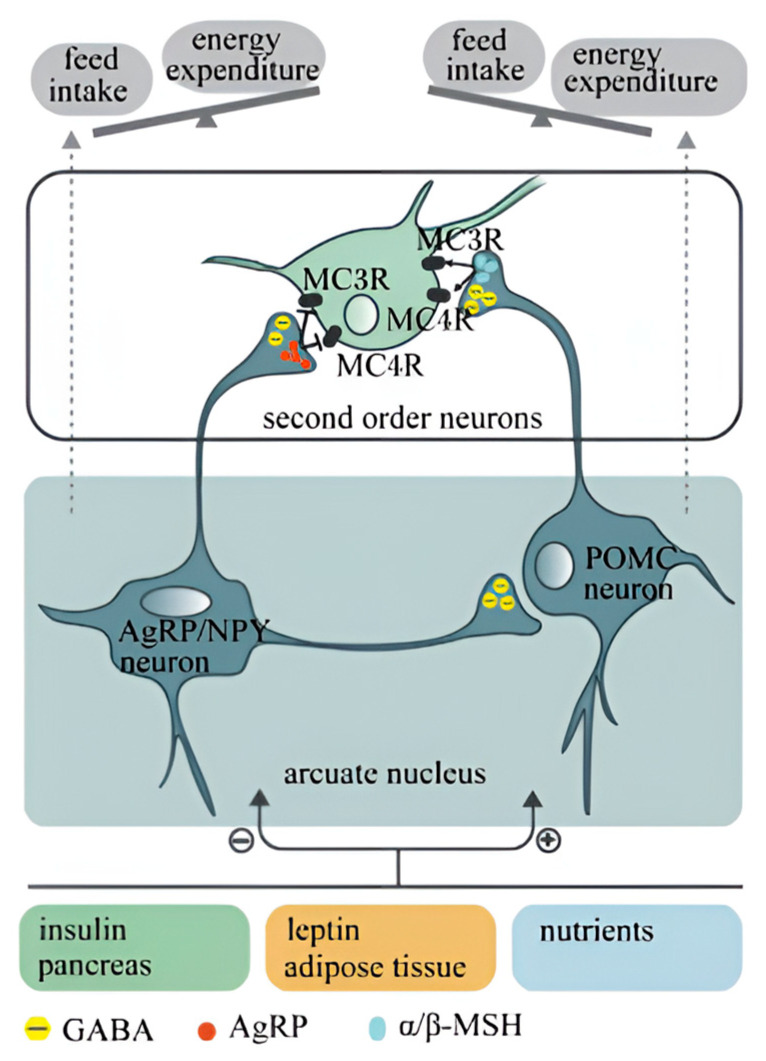
The hypothalamus regulates appetite pathways [38].

Feeding regulatory factors contribute to the hypothalamic regulation of appetite, hunger, and dietary behavior, classified as orexigenic (appetite-stimulating) or anorexigenic (appetite-suppressing) factors [28]. Orexigenic factors include neuropeptide Y (NPY), agouti-related peptide, and ghrelin. NPY, a neurotransmitter highly concentrated in the arcuate nucleus of the hypothalamus, inhibits satiety signals and stimulates appetite centers, thereby promoting feeding behavior; ghrelin, a gastrointestinal hormone, enhances appetite and feeding behavior by activating the hypothalamic hunger center [29]. Conversely, anorexigenic factors such as cholecystokinin (CCK), leptin, and insulin reduce appetite and suppress feeding [30,31]. These feeding regulatory factors modulate the appetite and dietary behavior of laying hens by influencing hypothalamic neural circuits and neurotransmission pathways. The interactions among these factors in the hypothalamus, along with their relationships with other neurotransmitters and hormones, collectively contribute to regulating the feeding behavior and feed efficiency of laying hens. The hypothalamus comprises both a feeding center and a satiety center [32]. The feeding center, located in the LHA, is the key region for stimulating feeding; the satiety center, primarily situated in the VMN, inhibits feeding by projecting its signals onto the feeding center to suppress its activity [33]. Peripheral feeding-related signals (such as insulin, leptin, glucose, etc.) arrive at the central nervous system, where they are processed and integrated in the hypothalamus [34]. The resulting responses either promote feeding via pathways involving NPY and AgRP or suppress feeding through pathways involving pro-opiomelanocortin (POMC) and cocaine-and-amphetamine-regulated transcript (CART) [35]. The blood–brain barrier within the arcuate nucleus of the hypothalamus senses changes in peripheral blood signals, comparing the strengths of appetite-stimulating and appetite-suppressing signals [36,37], thus integrating them to ultimately influence poultry feeding behavior. In conclusion, a comprehensive investigation into the physiological regulatory mechanisms of feeding in poultry is crucial for enhancing feed efficiency in laying hens (Figure 2).

### 2.2. Effects of Host Genetics on Feed Efficiency and Regulatory Mechanisms

Genetic factors play a pivotal role in determining the feed efficiency of laying hens [39]. Through selective breeding and genetic improvement programs, the identification and selection of strains or individuals exhibiting high feed efficiency can progressively enhance the overall feed conversion rate of the population [40]. These genetic factors significantly influence feed efficiency in laying hens through various mechanisms, including genetic background, genetic polymorphism, gene expression regulation, and gene interaction networks [41]. In recent years, there has been an increase in research focusing on the relationship between genetic background and feed efficiency in chickens. A deeper understanding of these genetic influences aids in the development of scientifically sound breeding strategies aimed at optimizing lineage composition and improving overall production performance.

Feed efficiency demonstrates moderate heritability [42], and substantial progress has been achieved in selecting for this trait over recent decades. Heritability (h^2^) quantifies the proportion of phenotypic variation in a quantitative trait within a population that is attributable to genetic factors [43]. In laying hen research, scientists frequently estimate the heritability of economically significant traits using pedigree or genomic data, providing a theoretical basis for subsequent genetic selection [44]. The measurement of feed conversion rates in laying hen populations commenced in the 1970s and has since evolved into a key breeding criterion, with individual feed efficiency assessments introduced as an additional evaluation method in the 1980s. Traditional selective breeding across multiple generations has resulted in a significant enhancement of laying hen feed efficiency. For example, the feed-to-egg ratio of white-shell laying hens decreased from 2.69 in the 1970s to 2.20 by the late 1990s [45] while the ratio for brown-shell laying hens declined from 2.86 to 2.18 [6].

The field of breeding has evolved significantly with scientific and technological advancements, transitioning to the molecular level. Molecular marker-assisted breeding, which utilizes DNA (or RNA) polymorphisms and employs tightly linked DNA molecular markers associated with target traits for selection, has become widely implemented. Common DNA molecular marker techniques include Restriction Fragment Length Polymorphism (RFLP), Random Amplified Polymorphic DNA (RAPD), DNA Fingerprinting (DAF), Amplified Fragment Length Polymorphism (AFLP), Simple Sequence Repeat (SSR), and Single-Nucleotide Polymorphisms (SNPs) [46]. Due to its efficiency, accuracy, and reliability, molecular marker-assisted breeding is extensively applied in laying hen breeding, primarily for quantitative trait locus (QTL) mapping, marker-assisted selection, genomic selection, and gene editing. QTLs refer to the locations of genes associated with specific quantitative traits [47]. Through molecular marker technologies, researchers can conduct genome scans in laying hens to identify QTLs linked to economically important traits. The localization of these QTLs enhances the understanding of the genetic loci that control traits in laying hens and provides valuable information about target genes for breeders. For example, Parsanejad et al. applied QTL technology in White Leghorn chickens to link the ornithine decarboxylase gene with residual feed intake at 35–38 weeks of age [48]. In the Hy-Line Brown layer strain [49], Research employed a 42K custom SNP chip platform and Bayesian association analysis to discover SNPs associated with feed intake and feed conversion efficiency across chromosomes 1, 2, 4, 7, 13, and Z. Additionally, LEPR and IGF are two critical genes related to growth, energy metabolism, and weight balance. Research using candidate gene approaches in laying hens has indicated that certain SNPs on LEPR may correlate with body weight, weight gain, feed intake, and feed conversion efficiency. Similarly, associational analyses have identified SNPs within the IGF gene that relate to growth rate, feed intake, feed efficiency, and fat deposition [50]. Genomic selection (GS) is a breeding decision-making method that utilizes whole-genome information [51]. By integrating molecular markers and phenotypic data, predictive models can be established to accurately estimate the breeding values of individuals. Wolc et al. simulated a laying hen breeding population and compared the effectiveness of genomic selection with conventional phenotypic selection, finding that offspring resulting from genomic selection exhibited superior advancements in egg weight, egg production, and production rates across 16 traits compared to those from conventional phenotypic selection [52]. Furthermore, when selecting for brown eggshell traits, both genomic selection and pedigree selection yielded similar genetic advancements; however, genomic selection reduced the generation interval by half [53]. The development of gene editing technologies, such as CRISPR-Cas9, presents new opportunities for laying hen breeding. Sex control technologies enable interventions in the reproductive processes of animals to produce offspring of the desired sex, which benefits poultry breeding by enhancing breeding efficiency and accelerating genetic improvement [54,55]. Consequently, there is an urgent need in the poultry industry for technologies that can control offspring sex through the expression of sex-determining genes (switch genes and regulatory elements). Currently, Israeli scientists have employed gene editing techniques to halt the development of male chicks within the egg prior to hatching, a breakthrough that could prevent the annual culling of billions of male chicks globally due to their inability to lay eggs [56]. Domestic research indicates that CRISPR technologies can directly and precisely modify, regulate, or silence the sex-determining genes in laying hens to maximize benefits [57,58]. In conclusion, the application of molecular marker-assisted breeding technologies in laying hen breeding not only enhances breeding efficiency and shortens breeding cycles but also makes the breeding process more precise and controllable. By utilizing these advanced technologies, laying hen breeders can more effectively select and cultivate birds with desirable traits, promoting continuous progress and development in laying hen breeding.

### 2.3. Effects of Nutritional Levels on Feed Efficiency and Regulatory Mechanisms

In contemporary farming practices within our country, inconsistent feed quality has resulted in a rapid decline in egg production rates following the peak laying period. Moreover, as laying hens age, their ability to absorb calcium and phosphorus from their diet diminishes, leading to an increased incidence of metabolic disorders and reduced secretion of reproductive hormones [59,60]. The primary cause of this issue is inadequate nutritional supply for the hens. The nutritional requirements of laying hens primarily comprise proteins, amino acids, carbohydrates, fats, energy, minerals, and vitamins derived from their feed [22]. These nutritional elements significantly influence the feed efficiency of laying hens by regulating enzyme activity, affecting feed digestion and absorption, participating in energy metabolism, and impacting physiological functions [61]. Optimizing the proportions and levels of nutritional elements in laying hen diets can enhance feed efficiency and improve economic outcomes in poultry farming. Appropriate nutritional components provide essential nutrients for maintaining vital functions and production, thereby promoting healthy growth and development. Research indicates that optimal growth and development contribute to improved feed efficiency in hens [62], as a well-developed digestive system can more effectively process and absorb nutrients from the feed.

In comparison to corn-based feed, wheat-based feed may decrease feed efficiency in chickens, primarily due to its higher content of indigestible soluble non-starch polysaccharides, which can potentially induce necrotic enteritis in chicks [63]. Moreover, developing scientifically sound and optimal feed formulations for laying hens has consistently been a central focus in animal nutrition research. In formulating diets for laying hens, the levels of energy and protein are paramount. For instance, regarding protein supply, modifying the proportions of various protein sources and amino acid ratios can mitigate excessive nitrogen excretion and ammonia emissions, thus enhancing the protein utilization efficiency of the hens [64]. Furthermore, appropriate levels of fats, carbohydrates, and fiber can influence energy utilization efficiency in laying hens. Suitable nutritional components contribute to improving the egg-laying performance of hens, including increased egg production rates [22], extended laying period [7], and enhanced egg quality [65]. Improved egg-laying performance correlates with greater economic benefits and feed efficiency. Consequently, to extend the laying cycle while maintaining production performance and feed efficiency during the extended laying period, optimizing feed formulations is a critical approach.

#### 2.3.1. Effects of Energy Levels on Feed Efficiency and Regulatory Mechanisms

Energy constitutes approximately three-quarters of poultry feed costs, making it a crucial factor in determining feed value [66]. Laying hens require energy for maintenance, growth, and egg production. Additionally, the metabolic processes of various nutrients in the feed depend on energy. Carbohydrates, fats, and proteins in the feed serve as the primary energy sources for laying hens. The metabolic energy system has evolved into a well-established method for assessing the energy value of poultry feed ingredients and is extensively utilized in poultry nutrition and feed formulation [67]. Under optimal housing conditions, laying hens require approximately 1.24 MJ/d of metabolic energy to produce one egg [68]. Excessive energy intake beyond the body’s actual requirements results in fat accumulation, leading to increased carcass fatness and reduced feed efficiency. When the dietary energy level reaches or exceeds 12.10 MJ/kg, it can impede the average daily weight gain of green-shelled layer chicks during the rearing period [69]. Research suggests that the optimal metabolic energy level for commercial layer chick feed can be reduced to 11.81 MJ/kg. For Taihang chicks during the rearing period, a suitable metabolic energy level is 11.50 MJ/kg, while “Jinghong No. 1” layer chickens require a metabolic energy level of approximately 11.70 MJ/kg during the rearing period for optimal results [70].

Fats represent a primary energy source in animal feed, possessing an energy value twice that of carbohydrates and proteins [71], making them a prevalent component in layer diets. Feed fats are primarily categorized into animal and plant-derived fats based on their origin. Plant fats, obtained through extraction or pressing, primarily include soybean, flaxseed, and corn oils [72]; Animal fats predominantly comprise fish oil and black soldier fly oil [73]. Poultry typically consume feed for energy, and in experiments designed to maintain equal energy and nitrogen levels, low doses of added fats (1–3%) generally do not significantly impact feed intake [74]. Research indicates that increasing linoleic acid supplementation enhances both the nitrogen deposition rate and nitrogen-corrected apparent metabolic energy of the feed, consequently improving feed conversion rates [75]. Furthermore, appropriate fat consumption can enhance fatty acid oxidation, maintaining stable blood glucose levels and suppressing appetite. The addition of fats can also reduce the heat increment of feed [76], suggesting that higher fat doses may decrease feed intake in laying hens without compromising egg production performance, thus enhancing feed efficiency. In a study conducted by Kishore et al. [77], supplementing laying hen diets with 3% flaxseed oil did not significantly affect egg production rates compared to the control group without added fats. However, it notably reduced feed intake and the feed-to-egg ratio, thereby improving feed efficiency.

Laying hens require an adequate supply of fat to maintain their egg production performance, with the majority of this fat synthesized in the liver [2]. To sustain high production levels, laying hens must secrete substantial amounts of estradiol during egg production to support follicle development, as elevated levels of estradiol promote fat synthesis and accumulation in the liver [78]. The fats synthesized in the liver need to be transported via low-density lipoproteins, which predisposes laying hens to lipid metabolism disorders [79], potentially resulting in decreased production performance and significantly reduced feed efficiency. For instance, incorporating 6% canola oil into the diet of laying hens can decrease egg production rates, average egg weights, and average daily feed intake [80]. Consequently, the judicious addition of an appropriate amount of fat in laying hen diets can effectively enhance production performance and feed efficiency, thereby improving economic benefits.

#### 2.3.2. Effects of Crude Protein Levels on Feed Efficiency and Regulatory Mechanisms

In laying hen nutrition research, protein-related studies primarily concentrate on the equilibrium between protein and energy, the implementation of low-protein diets, and the exploration of novel feed ingredients. While protein is not typically considered an energy source, insufficient dietary energy can lead to protein conversion into energy, increasing physiological stress and resulting in protein wastage [22]. Conversely, excessive dietary energy levels can decrease feed intake in laying hens, consequently reducing the consumption of protein and other essential nutrients, thereby impacting production efficiency and feed utilization [81]. Thus, maintaining an appropriate protein-to-energy ratio is crucial for formulating optimal laying hen feed. Research suggests that the ideal crude protein level for Taihang chicken during the growth phase is 14.00–15.00% [82].

China currently faces significant challenges regarding the scarcity of protein feed resources (such as soybeans) and nitrogen emissions pollution during the breeding process [83]. Low-protein diets, also known as low-protein amino acid-balanced diets, have a crude protein content that is reduced by 2–4 percentage points compared to conventional diets, while synthetic amino acids are added to meet the amino acid requirements of poultry, enhancing protein utilization precision and effectiveness. These diets can significantly improve feed protein utilization, offering advantages such as cost savings, reduced nitrogen emissions, the ease of application, and stable performance, thus presenting broad application prospects. Research by Burley et al. on Roman hens aged 18–51 weeks demonstrates that low-protein and medium-protein diets are less costly than high-protein diets without affecting egg production, resulting in higher economic benefits [84]. Therefore, implementing low-protein amino acid-balanced diets can provide substantial economic benefits for commercial laying hen populations, provided that production performance is maintained. Some studies suggest that ensuring the addition of a 0.72% combination of Met and Cys in low-protein diets can achieve optimal production results [85]. As crude protein concentration decreases, the content of non-essential amino acids also decreases; consequently, it is necessary to increase the supply of essential amino acids, represented by Lys, to maintain normal production performance. Amino acid deficiency in laying hens can lead to changes in gut microbiota and is closely related to key physiological and production parameters (such as body weight, abdominal fat, feed intake, egg production rate, egg weight, and feed utilization rate). Therefore, essential amino acids should be added in appropriate amounts to low-protein diets, particularly Met, Lys, Ile, and Thr [86]. However, it should be noted that the technology for low-protein diets in laying hen rearing is still in the exploratory stage, and the long rearing cycle of laying hens is influenced by various factors. Nutrition during the chick or growing stages may also affect subsequent egg production; thus, the reduction in protein levels in laying hen diets should be moderate. For instance, Wang et al. found that supplementing crystalline amino acids in low-protein diets suppressed feed intake and lipoprotein synthesis in laying hens, thereby reducing egg production performance [30]. Consequently, future research needs to delve deeper into the balance between protein, energy, and amino acids in diets.

#### 2.3.3. Effects of Trace Elements on Feed Efficiency and Regulatory Mechanisms

In poultry production, trace elements are essential feed supplements that significantly influence the production performance of laying hens and egg quality. These trace elements are primarily classified into inorganic and organic forms. Recent research on mineral elements in poultry feed has predominantly focused on the nutritional requirements and feed efficiency of calcium, phosphorus, manganese, zinc, iron, selenium, and other elements [87]. Inorganic trace elements (ITEs), such as copper, iron, zinc, and manganese, are extensively utilized in animal production due to their cost-effectiveness. However, numerous studies have demonstrated that the bioavailability of ITEs is relatively low. Research indicates that poultry exhibit poor utilization rates for heavy metal elements like copper, iron, zinc, and manganese in their diets, with over 90% of added trace elements being excreted through manure, resulting in environmental pollution and feed resource wastage [88,89].

Studies have demonstrated that organic trace elements (OTSs) exhibit higher bioavailability compared to inorganic trace elements (ITSs) [90]. The appropriate addition of OTSs to diets can enhance eggshell quality and its microstructure [91]. OTSs, formed through the chelation of minerals and organic compounds, can act more effectively at lower concentrations while reducing excretion levels [88], thereby minimizing environmental pollution and improving feed utilization efficiency. Research has revealed that certain organic acids can undergo polymerization under specific conditions, acting as chelating agents that bind to trace elements to form OTSs. This process can effectively enhance the production performance and feed efficiency of laying hens during the later stages of egg production and improve egg quality [92,93]. The underlying principle is that trace elements chelated with amino acids become more soluble and easier to transport within the body, leading to improved absorption compared to their inorganic counterparts. In addition to replacing ITSs with OTSs, other forms of trace elements, such as those utilizing nanotechnology, yeast protein salts, and fermented forms, have been investigated and applied. Research indicates that incorporating fermented forms of trace elements into diets positively impacts the production performance of laying hens and can effectively reduce environmental pollution caused by their manure [94].

### 2.4. Effects of Environmental Factors on Feed Efficiency and Regulatory Mechanisms

The environmental conditions within a poultry house, including temperature, lighting, humidity, and ventilation, directly influence the feed efficiency of chickens. Chickens lack sweat glands and primarily regulate their body temperature through respiration [95]. Consequently, appropriate temperature and humidity levels are crucial for the normal growth and metabolism of laying hens, which in turn enhances feed efficiency. When temperatures and humidity levels in the poultry house exceed optimal ranges, the metabolic heat load on the hens increases, potentially disrupting carbohydrate and protein metabolism. This disruption can result in a decrease in the digestion and nutrient absorption capacities of the feed, thereby reducing overall feed efficiency.

Lighting plays a crucial role in influencing laying hens, as it regulates their circadian rhythms, feeding behavior, and metabolic processes [96]. Optimal lighting conditions can enhance normal feeding and digestive functions in hens, thereby improving feed utilization efficiency. In comparison to conventional commercial lighting schedules (18 h of light and 6 h of darkness), intermittent lighting has the potential to extend the retention time of food in the crop, which may contribute to improved feed efficiency.

Air quality plays a crucial role; the concentrations of pollutants such as ammonia, carbon dioxide, and particulate matter directly affect feed efficiency in laying hens [97]. Elevated levels of ammonia and carbon dioxide can impair the respiratory and intestinal functions of the hens, resulting in reduced digestion and nutrient absorption efficiency.

Furthermore, appropriate space allocation and stocking density significantly influence the behavioral patterns and physiological status of chickens [98]. Adequate space facilitates normal movement and feeding behavior in hens, thereby enhancing feed efficiency. Conversely, excessive stocking density may elevate competition and stress among chickens, subsequently affecting feed digestion and nutrient absorption [99]. For free-range chickens, the choice of bedding materials and flooring type also impacts production performance [100]. In conclusion, environmental factors play a crucial role in the feed efficiency of laying hens. Maintaining suitable temperature and humidity levels, providing quality lighting, ensuring good air quality, allocating sufficient rearing space, and offering a well-designed free-range environment can enhance feed conversion efficiency, ultimately increasing the economic viability of poultry farming.

### 2.5. Effects of Exogenous Additives on Feed Efficiency and Regulatory Mechanisms

#### 2.5.1. Probiotics

Probiotics have gained prominence due to their capacity to significantly improve laying hen performance. However, concerns regarding antibiotic resistance and drug residues led China to implement regulations on antibiotic usage beginning in 2020 [101]. Following this restriction, health and disease challenges in poultry production have become more pronounced, particularly affecting the intestinal tract. Various intestinal disorders, including viral enteritis (caused by reoviruses, infectious bursal virus, etc.), bacterial enteritis (attributed to *Clostridium perfringens*, *Escherichia coli*, *Salmonella*, etc.), and parasitic infections (such as coccidiosis), have emerged, underscoring the pressing need for effective and economically viable alternatives [102].

Presently, the production of laying hens primarily employs probiotics, prebiotics, enzyme preparations, and other substitutes [103]. Probiotics are defined as live microorganisms that beneficially support the balance of intestinal flora in humans or animals [104]. They predominantly originate from normal physiological bacteria and non-intestinal bacteria found within the animal intestine. Common probiotics utilized in laying hen farming include species such as *Bacillus*, *Lactobacillus*, *Bifidobacteria*, *Clostridium butyrate*, and *yeast*. Probiotics are favored alternatives due to their protective effects through various positive actions. The mechanisms by which probiotics exert their effects commence with the establishment of a mucosal barrier [105]. As dominant members of the normal intestinal flora, probiotics adhere closely to the intestinal mucosa, forming a biological barrier that helps maintain microbial balance and mitigates the impact of inflammatory cytokines. Furthermore, probiotics possess antibacterial properties; they suppress the growth of potential pathogens in the intestine by lowering pH levels, preventing bacterial adhesion to intestinal epithelial cells, and minimizing bacterial translocation within the intestinal lumen [106]. Lastly, probiotics play a role in modulating the anti-inflammatory and pro-inflammatory responses of innate immunity, thereby enhancing the immune response and promoting antibody formation [107].

Research suggests that incorporating an optimal proportion of probiotics into feed can rapidly enhance disease resistance in laying hens and improve their digestive and absorptive capacities, leading to increased feed utilization efficiency [108]. For example, lactic acid bacteria, beneficial microorganisms that ferment to produce lactic acid, promote chicken growth and development, mitigate stress responses, and enhance feed efficiency by establishing a balanced intestinal microecological environment [109]. Bacillus subtilis, an anaerobic spore-forming bacterium, creates a biochemical barrier within the chicken intestine. This barrier strengthens intestinal defense against pathogens, enhances immune function, reduces stress responses, provides nutritional benefits, and facilitates chicken growth and development, ultimately improving production efficiency and feed quality [110].

#### 2.5.2. Enzymes

In poultry production, the absence of specific digestive enzymes in avian species, coupled with the presence of anti-nutritional factors in feed, frequently results in the incomplete digestion, absorption, and utilization of nutrients. This inefficiency can lead to suboptimal nutrient utilization, increased feeding costs, reduced feed availability, and diminished economic returns. Research has demonstrated that incorporating targeted enzyme preparations into feed can effectively counteract these anti-nutritional factors, thereby enhancing nutrient digestion and absorption [111]. This supplementation significantly improves the utilization rates of essential nutrients, particularly protein and starch, expands the range of viable feed ingredients, and contributes to lower feed costs. Furthermore, the application of enzyme preparations can reduce the moisture content and nutrient levels in poultry excreta, promoting gastrointestinal health in laying hens and mitigating environmental pollution [112].

Chickens possess relatively short digestive tracts, which often lead to challenges such as incomplete nutrient digestion and absorption, as well as an increased viscosity of intestinal chyme, impeding nutrient assimilation [113]. Consequently, incorporating enzymes into their diet is recommended. Current research on the application of feed enzyme preparations in laying hen diets is comparatively limited relative to studies on broilers. Scholey et al. demonstrated that enzyme supplementation in layer feed enhances nutrient digestion and absorption, improves feed conversion efficiency, and increases egg production performance [114]. Dänicke et al. observed that supplementing corn–soybean meal diets of laying hens with compound enzymes (primarily xylanase, β-glucanase, and vibrinose) significantly improved feed conversion rates [115]. Similarly, Mathlouthi et al. reported that adding compound enzyme preparations (mainly containing xylanase and β-glucanase) to a wheat–soybean meal basal diet for laying hens significantly enhanced feed conversion rates, without notable changes in egg production rate or egg weight [116]. Liu et al. (1998) noted that while supplementing barley diets with crude enzyme preparations did not significantly increase egg-laying rates, it did improve egg quality [117]. In another study, Liu et al. reported significant increases in egg production rates (5.39% to 7.47%), reductions in soft-shelled egg rates (3.24% to 3.91%), and improvements in feed–egg ratios (decreasing by 2.24% to 14.38%) when incorporating compound enzyme preparations into the corn–soybean meal diet for Roman laying hens [118]. He et al. found that while the compound enzyme preparation did not significantly affect the overall production performance of laying hens, it markedly improved eggshell quality and mitigated the decline in egg production during later laying stages [119]. These findings collectively suggest that enzyme supplementation in laying hen diets can enhance egg-laying performance, feed conversion efficiency, and the utilization of energy and egg white. However, the current application of enzyme preparations in laying hen production remains limited, underscoring the need for further research and exploration in this area.

### 2.6. Effects of Hormonal Regulation on Feed Efficiency and Regulatory Mechanisms

Hormones play a pivotal role in regulating feed utilization efficiency in laying hens. Their primary mechanism of action involves binding to receptors in the cell nucleus, thereby modulating gene expression and influencing essential physiological processes. These processes include metabolism, growth and development, nutrient absorption, and energy metabolism. Consequently, hormones directly or indirectly affect the efficiency with which laying hens utilize nutrients from their feed [31]. Key hormones involved in this regulatory process include growth hormone, thyroid hormones, and insulin.

Growth hormone, a protein hormone secreted by the anterior pituitary gland, plays a crucial role in promoting growth and development in laying hens. This hormone stimulates the synthesis and secretion of digestive enzymes in the gastrointestinal tract, enhancing the degradation and absorption efficiency of feed. It also promotes the proliferation and differentiation of intestinal epithelial cells, increasing the absorptive surface area of the intestine, which enhances the capacity for feed digestion and absorption [120]. Moreover, growth hormone modulates energy metabolism in laying hens, increasing the uptake and utilization of glucose in the gastrointestinal tract, facilitating its conversion and utilization in the liver and other tissues, thereby improving energy utilization efficiency [31]. Additionally, growth hormone promotes the breakdown of adipose tissue, increasing the release and utilization of fatty acids, which enhances fatty acid oxidation and energy production, significantly improving feed utilization efficiency [121]. Insulin-like growth factors (IGFs), a group of polypeptide hormones closely associated with growth hormone, regulate protein synthesis and cell proliferation in laying hens through various mechanisms [122], impacting feed utilization efficiency. IGFs directly stimulate protein synthesis within the hens [123] by activating the mTORC1 signaling pathway within cells, promoting protein synthesis reactions in hen tissues and enhancing the utilization efficiency of protein in the feed. Furthermore, IGFs indirectly influence cell proliferation by regulating biological processes such as the cell cycle and DNA synthesis [24], thereby affecting feed utilization efficiency. Thyroid hormones, primarily secreted by the thyroid gland, include triiodothyronine (T3) and thyroxine (T4) [124]. These hormones affect energy metabolism and feed utilization efficiency in laying hens by increasing the rate of oxidative metabolism, altering energy distribution, and promoting protein synthesis. Thyroid hormones increase the rate of oxidative metabolism in laying hens, raising the basal metabolic rate (BMR) [125]. This leads to an increase in the basic energy consumption of the hens, thereby enhancing feed utilization efficiency. Additionally, thyroid hormones influence the distribution of energy within the hens, facilitating the conversion and utilization of glucose between the liver and other tissues, accelerating the oxidation of fatty acids, and reducing fat deposition [126], thus improving energy utilization efficiency. Furthermore, thyroid hormones affect protein utilization by regulating protein synthesis and degradation processes [127].

In laying hen production, hormonal regulation is primarily influenced by dietary composition and management practices. Research demonstrates that feed nutrient composition significantly affects hormone secretion. Appropriate protein levels and amino acid intake can enhance hormone synthesis and secretion, thereby improving feed utilization efficiency. For example, incorporating suitable levels of methionine, iodine, and selenium into laying hen diets is essential for thyroid hormone synthesis and secretion [128]. Additionally, feeding frequency and timing can modulate the secretion rhythm of growth hormone from the pituitary gland [129]. Through strategic feeding management, such as increased feeding frequency and reduced nocturnal feeding, growth hormone levels can be elevated, subsequently improving feed utilization efficiency [130]. Consequently, a comprehensive understanding of hormonal regulatory mechanisms in laying hens can facilitate the optimization of management practices, enhance feed utilization efficiency, reduce production costs, and improve overall productivity.

### 2.7. Effects of Health Status on Feed Efficiency and Regulatory Mechanisms

The health status of hens significantly impacts their immune system, which can substantially affect production performance and feed utilization efficiency [131,132]. Infectious diseases represent the primary concern among factors influencing the health and productivity of laying hens. Compromised immune function and disease resistance in hens can result in decreased appetite and impaired digestion and absorption, leading to feed resource wastage and reduced feed efficiency. Common infectious diseases in poultry farming include avian influenza, Newcastle disease, infectious laryngotracheitis, and infectious bronchitis. Avian influenza, a highly contagious viral disease, is categorized into highly pathogenic avian influenza (HPAI) and low pathogenic avian influenza (LPAI) [133]. Both virus types can compromise the hens’ immune system, causing respiratory symptoms, gastrointestinal disturbances, and decreased egg production. Newcastle disease, caused by the Newcastle disease virus, is another highly contagious disease. Infected hens may exhibit respiratory, neurological, and gastrointestinal symptoms, potentially leading to reduced egg production and quality, as well as increased flock mortality [134]. Infectious bronchitis, a respiratory disease caused by a coronavirus, results in respiratory symptoms and tracheal and pulmonary lesions, ultimately reducing egg production and quality [135]. Consequently, maintaining laying hen health and implementing effective disease prevention and control measures are crucial for enhancing feed utilization efficiency.

### 2.8. Effects of Microbial Community on Feed Efficiency and Regulatory Mechanisms

#### 2.8.1. The Composition of the Digestive Organs of Laying Hens

**Figure 3:**
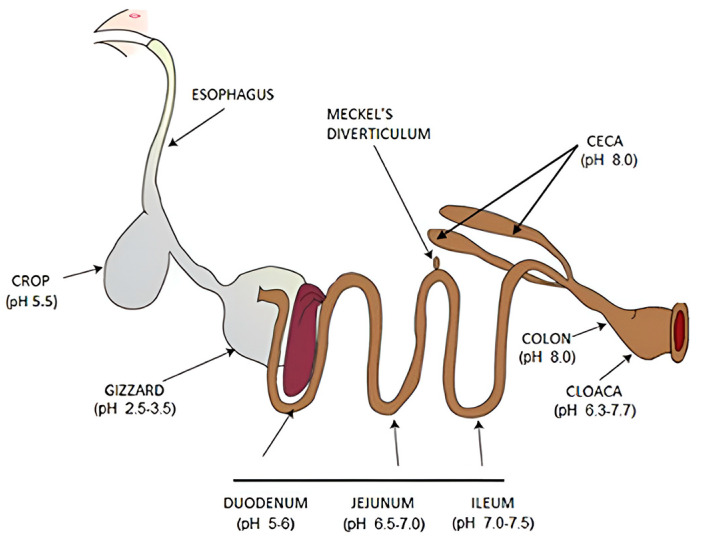
Major organs of the gastrointestinal tract in chicken [142].

The digestive system of laying hens is characterized by its relative brevity and comprises the oral cavity (Figure 3), esophagus, crop (bursa sac), gizzard (muscular stomach), proventriculus (glandular stomach), duodenum, jejunum, ileum, cecum, rectum, and cloaca [136]. The digestive process commences when feed enters the oral cavity. Salivary glands secrete mucus, facilitating the transfer of feed from the mouth to the esophagus. As the feed progresses through the esophagus, hydrochloric acid and pepsin are released, initiating protein digestion. Subsequently, peristaltic movements propel the feed either into the crop or directly into the stomach. The complete transit of feed through the digestive tract typically requires approximately 3 to 4 h [137].

The avian stomach in laying hens comprises two distinct regions: the glandular stomach and the muscular stomach [138]. The glandular stomach secretes pepsin and hydrochloric acid, facilitating chemical digestion. In contrast, the muscular stomach is responsible for mixing and grinding feed. When the muscular stomach is empty, feed bypasses the bursa; however, when full, the bursa serves as a storage site for feed [139]. Following mechanical processing in the muscular stomach, the feed progresses to the small intestine, which consists of the duodenum, jejunum, and ileum—the primary sites of digestion and nutrient absorption. Subsequently, the feed enters the double-ended cecum, where starch, cellulose, and indigestible carbohydrates undergo fermentation and further digestion. The remaining material then passes from the rectum to the cloaca, where it combines with uric acid before excretion [140,141].

#### 2.8.2. Impact of Gut Environment on Feed Efficiency

##### Influence of Gut Villus Structure on Feed Efficiency

The small intestine plays a pivotal role in food digestion and nutrient absorption, particularly amino acids and carbohydrates, making it integral to improving feed efficiency in organisms [143]. Key morphological parameters that reflect gut function include the length of gut villi and the depth of crypts. Generally, longer villi and shallower crypt depths indicate a higher maturity of intestinal epithelial cells, leading to the accelerated synthesis and secretion of digestive enzymes, thus enhancing the intestine’s digestive capacity [144]. Research conducted by Yasar et al. demonstrates that wet feeding can positively influence villus height and structure, thereby affecting feed efficiency [145]. While the digestive and absorption capacity of the intestine is closely linked to its physiological state, further investigation is necessary to determine whether differences in high and low feed conversion rates stem from histological variations in the intestine.

##### Influence of Gut Microbiota on Feed Efficiency

As research on the gut environment has progressed, scientists have increasingly recognized that an animal’s genome encompasses not only the host’s own genetic material but also the gut microbiome. While the host’s genomic composition remains relatively stable, the gut microbial genome exhibits dynamic properties. The gut microbiota undergoes changes in response to probiotic consumption or environmental alterations. Moreover, the gut microbiota can modify its genomic composition to adapt to diverse environments by modulating the abundance of specific microbes and incorporating new microbial species [146,147].

The gut microbiota, a crucial symbiotic microbial community for the host, actively participates in various physiological functions, including nutrient digestion and absorption, metabolism, gut barrier integrity, and mucosal immunity. Recent studies have confirmed the influence of the gut microbiota on the nutrition, health, physiology, immunity, and production performance of chickens [148]. In layer hen research, advancements in high-throughput sequencing technologies have enabled researchers to elucidate the mechanisms by which gut microbiota affect nutrient utilization, gut barrier formation, production performance, and egg quality. Furthermore, gut microbes are intricately linked to the host’s immunity, metabolism, and behavior through the regulation of axes such as the gut–liver and gut–brain axes [149]. Gut microbes and their metabolites function as signaling molecules, connecting the gut with the liver, brain, and reproductive systems, thereby exerting direct or indirect effects on poultry health and egg quality. Consequently, variations in the composition of the host’s microbiota significantly impact feed efficiency.

Research employed terminal restriction fragment length polymorphism (T-RFLP) to examine microbiota communities in the ileum and ceca of chickens with varying feed efficiencies. Their findings revealed higher abundances of *Clostridium lactatifermentans*, *Gallibacterium anatis*, and *Escherichia coli* in individuals with low feed conversion rates, while *Lactobacillus* species were more prevalent in those with high conversion rates [150]. Yuan et al. conducted 16S RNA sequencing on the duodenum, ceca, and fecal microbiota of layer hens with high and low feed efficiencies, identifying significant differences in the cecal microbiota [113]. In chickens with high feed efficiency, the relative abundances of Lactobacillus and *Akkermansia* were elevated [151]. Furthermore, PIHCUSt analysis indicated that the microbiota in the high feed efficiency group exhibited enrichment in pathways associated with amino acid and carbohydrate metabolism; meanwhile, PIHCUSt analysis indicated that the microbiota in the high feed efficiency group was enriched in pathways related to amino acid and carbohydrate metabolism [152].

**Figure 4:**
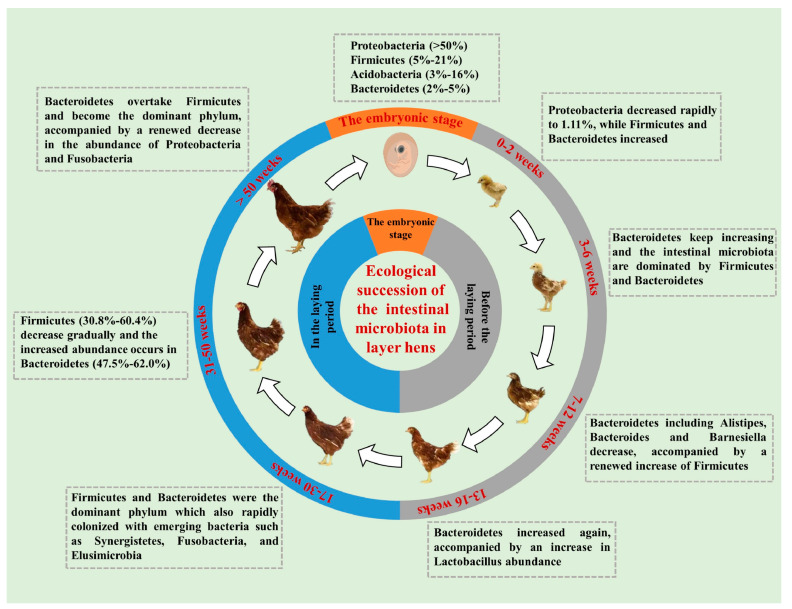
Microbial compositions at different stages of laying hens [153]. This cycle illustrates dynamic microbiota shifts (e.g., *Firmicutes–Bacteroidetes* balance) across layer hens’ life stages, linked to digestive function and feed efficiency.

The gut microbial community of layer hens represents a dynamic ecosystem that continually adapts to the individual’s physiological requirements (Figure 4). Throughout the laying period, the composition of the gut microbiota in hens undergoes corresponding alterations [153]. During this time, Firmicutes, Bacteroidetes, and Proteobacteria are considered the primary dominant microbes, with their relative abundances fluctuating as the hens age [154]. In the early laying stage, endocrine and sex hormone changes may influence the gut microbiota composition [155]. During this phase, Firmicutes predominate but their abundance decreases while Bacteroidetes abundance increases. In the peak laying period, the abundance of Firmicutes gradually declines, while Bacteroidetes abundance rises [156]. By the late laying period, the relative abundance of Bacteroidetes surpasses that of Firmicutes, becoming the predominant community [157]. Research observed that the cecal microbiota of 1- to 28-day-old White Leghorn chicks developed rapidly, with Proteobacteria dominating at over 85% abundance from days 1–3. The abundance and taxonomic diversity of Firmicutes began to increase around day 7 and stabilized above 85% after day 14 [158]. Ngunjiri et al. found that the dominant bacteria in the ileum of layer hens aged 1–51 weeks were all *Lactobacillus* species, including *Lactobacillus curvatus*, *Lactobacillus johnsonii*, and *Lactobacillus reuteri*, while the abundance of Clostridium gradually increased starting from the early laying stage [159].

The colonization of the gut microbiota in layer hens is influenced by genetic background, feed additives, and environmental factors. Maintaining the diversity of beneficial microbial communities and the microecological balance in the gut is crucial for ensuring normal physiological functions. Studies examining fecal microbiota from high- and low-fat hens have revealed significant differences in the gut microbiota among groups [160]. Additionally, research on Roman brown and Roman LSL-Classic layer hens demonstrated that breed had a substantially greater impact on the gut microbiota compared to feed additives [118]. These findings suggest that genetic background and phenotypic differences between chicken breeds and lines may regulate gut microbial composition by influencing nutrient metabolism and gastrointestinal development, although the specific mechanisms require further investigation. The levels of calcium and phosphorus in feed significantly affect the abundance of *Prevotellaceae, Methanobacteriaceae, Lactobacillaceae, Megasphaera, Bacteroidaceae, Spirochaetaceae, and Streptococcaceae* in layer hens’ intestines, which are closely related to gut health, production performance, and immune system regulation [161]. Furthermore, changes in feeding environment and methods can also influence the composition of gut microbiota in layer hens [154]. For instance, environmental stress may reduce feed intake in poultry, affecting the permeability of the intestinal mucosa and the structure of the microbial community, potentially leading to intestinal inflammation and decreased production performance [162]. While extensive research has been conducted on the effects of gut microbiota on the feed efficiency of layer hens, experimental samples remain limited. Further investigations in larger populations of layer hens are necessary to provide a more comprehensive reference for exploring the relationship between feed efficiency and microbiota.

## 3. The Significance of Modern Molecular Biology Techniques

In the contemporary landscape of rapidly advancing molecular technologies, multi-omics approaches have emerged as potent tools for elucidating molecular mechanisms related to feed conversion efficiency in laying hens. These methodologies encompass genomics, transcriptomics, proteomics, and metabolomics, among others [163]. By leveraging these technologies, researchers can generate comprehensive datasets on gene expression, protein composition, and metabolites, thereby enhancing our understanding of the molecular mechanisms influencing feed conversion efficiency in laying hens. For instance, the regulatory mechanism of the gut–liver axis holds significant importance in the study of feed utilization efficiency.

Genomic studies are instrumental in identifying key genes involved in feed digestion and metabolism in laying hens [164]. The sequencing of the laying hen genome and annotation of gene functions enable the identification of genes associated with feed conversion efficiency, facilitating the further exploration of their roles in feed metabolism. For example, specific genetic mutations or variations in gene expression may be observed in hens exhibiting high feed conversion efficiency, potentially influencing their energy and nutrient utilization [165]. Transcriptomic analyses provide insights into gene expression patterns in laying hens [166]. An examination of transcriptomes from tissues such as the gut and liver can reveal gene expression differences linked to feed digestion, absorption, and metabolism. This information enhances the understanding of regulatory mechanisms governing gene expression in laying hens under various feed conditions and highlights strategies to improve feed efficiency through gene expression modulation. Research has identified a significant correlation between the SNP locus of the SLC5A1 (sodium–glucose co-transporter) gene and feed efficiency (FE) through genome-wide association studies (GWAS), with Han sheep carrying the dominant allele showing an 8% increase in FE [167]. Proteomics is crucial in investigating protein composition and abundance alterations in laying hens [168]. A comparison of protein profiles between hens with efficient and inefficient feed conversion can identify critical proteins associated with feed efficiency [169]. These proteins may play significant roles in essential processes such as feed digestion, nutrient absorption, and energy metabolism. Further investigation into the functions and regulatory mechanisms of these proteins can yield valuable insights for improving feed conversion efficiency. Metabolomics provides essential information regarding metabolic byproducts in laying hens. An analysis of metabolites present in serum, urine, or feces offers a clearer picture of the metabolic status of laying hens under different feeding regimes [170]. Correlating this data with feed conversion efficiency allows the identification of specific metabolic pathways and metabolites closely linked to feed utilization efficiency [171]. Such insights deepen the understanding of energy and nutrient utilization patterns in laying hens across various feeding conditions, providing a theoretical foundation for enhancing feed efficiency. In conclusion, multi-omics technology holds significant importance in studying the feed conversion efficiency of laying hens. By elucidating molecular mechanisms, identifying biomarkers, enhancing feed utilization, and optimizing feed formulation and management strategies, these technologies can substantially contribute to the economic viability and sustainable development of layer production. It is worth noting that we have summarized the influences affecting FCE in the article (Table 1).

## 4. Conclusions

Feed utilization efficiency in laying hens remains a linchpin for balancing economic viability and environmental sustainability in poultry production. This review has synthesized key determinants of feed efficiency—genetics, nutrition, environment, disease, and the gut microbiota—along with regulatory mechanisms underpinning efficiency improvements. Looking forward, future research should prioritize integrating multi-omics approaches to unravel gene–environment interactions and mine functional genes governing feed efficiency. Challenges persist in deciphering complex microbial–host metabolic crosstalk and translating laboratory findings into cost-effective field applications. Technological innovations like genomic selection, CRISPR-based gene editing, and precision nutrition modeling will expedite genetic progress and dietary optimization. Importantly, advancing feed efficiency must align with sustainability goals: reducing nitrogen/phosphorus excretion, minimizing feed-related carbon footprints, and developing eco-friendly feed additives. By fostering interdisciplinary collaboration across molecular biology, nutrition science, and environmental engineering, the industry can achieve dual objectives of enhancing feed conversion ratios and promoting low-carbon, resource-efficient laying hen production.

## Figures and Tables

**Table 1:** The main factors affecting the feed utilization efficiency of laying hens.

Influencing Factors	Major Impacts	Author/s
Feeding Behavior	Feeding behavior impacts laying hens’ feed efficiency via feed intake, influenced by genetics/nutrition, with both low/high intake harming efficiency. Regulated by hypothalamic neural networks via orexigenic/anorexigenic factors, studying mechanisms enhances feed efficiency.	[22,23,24,25,26,27,28,29,30,31,32,33,34,35,36,37]
Host Genetics	Genetic factors significantly influence laying hens’ feed efficiency via selective breeding and advanced technologies (molecular markers, genomic selection, and gene editing), enhancing feed conversion and breeding efficiency.	[39,40,41,42,43,44,45,46,47,48,49,50,51,52,53,54,55,56,57,58]
Nutritional Levels	In modern farming, inconsistent feed quality and aging hens’ reduced nutrient absorption—caused by inadequate nutrition—and impaired feed efficiency via disrupted enzyme activity and metabolism. Optimizing dietary energy, protein, and trace elements (e.g., organic forms over inorganic) enhances digestion, egg-laying performance, and feed conversion, while wheat-based feeds reduce efficiency due to indigestible polysaccharides.	[59,60,61,62,63,64,65,66,67,68,69,70,71,72,73,74,75,76,77,78,79,80,81,82,83,84,85,86,87,88,89,90,91,92,93,94]
Environmental Factors	Environmental factors (temperature, humidity, lighting, air quality, and stocking density) directly impact laying hens’ feed efficiency. Suboptimal conditions disrupt metabolism, impair digestion, and reduce nutrient absorption, while suitable environments enhance feed conversion and economic viability.	[95,96,97,98,99,100]
Exogenous Additives	Exogenous additives (probiotics and enzymes) enhance laying hens’ feed efficiency by regulating gut flora and improving nutrient digestion. Probiotics replace antibiotics post 2020 restrictions, while enzymes counter anti-nutritional factors, though enzyme application needs more research.	[101,102,103,104,105,106,107,108,109,110,111,112,113,114,115,116,117,118,119]
Hormonal Regulation	Hormones like growth hormone, thyroid hormones, and insulin regulate feed efficiency in laying hens by modulating gene expression, digestion, nutrient absorption, and energy metabolism. Growth hormone enhances digestive enzyme secretion and intestinal absorption, while IGFs promote protein synthesis via mTORC1. Thyroid hormones (T3/T4) boost oxidative metabolism and energy utilization. Dietary composition (protein and amino acids) and feeding management (frequency and timing) influence hormone secretion, optimizing feed efficiency and productivity.	[31,120,121,122,123,124,125,126,127,128,129,130]
Microbial Community	The gut microbiota of laying hens influences feed efficiency via nutrient digestion, metabolism, and gut immunity, with high-efficiency groups having more beneficial bacteria like Lactobacillus, affected by genetics, feed, and environment.	[136,137,138,139,140,141,142,143,144,145,146,147,148,149,150,151,152,153,154,155,156,157,158,159,160,161,162]
Modern Molecular Biology Techniques	Multi-omics technologies (genomics, transcriptomics, proteomics, and metabolomics) aid in elucidating molecular mechanisms of feed conversion efficiency in laying hens, identifying key genes/proteins/metabolites to optimize feed utilization and promote sustainable poultry production.	[163,164,165,166,167,168,169,170,171]
