# Correction: Gao et al. Molecular Mechanisms and Regulatory Factors Governing Feed Utilization Efficiency in Laying Hens: Insights for Sustainable Poultry Production and Breeding Optimization. *Int. J. Mol. Sci.* 2025, *26*, 6389

**DOI:** 10.3390/ijms27209024

**Published:** 2026-10-12

**Authors:** Zhouyang Gao, Jiangxia Zheng, Guiyun Xu

**Affiliations:** College of Animal Science and Technology, China Agricultural University, Beijing 100193, China; gaozhouyangcau@163.com

In the original publication [1], there was a mistake in Figure 1 as published. During the post-publication review, the authors identified a potential copyright and attribution issue concerning the figure. The authors decided to remove Figure 1 from the article to avoid any potential copyright or attribution concerns. The authors state that the scientific conclusions are unaffected. This correction was approved by the Academic Editor. The original publication has also been updated.
